# CDSANet: A CNN-ViT-Attention Network for Ship Instance Segmentation

**DOI:** 10.3390/jimaging11110383

**Published:** 2025-10-31

**Authors:** Weidong Zhu, Piao Wang, Kuifeng Luan

**Affiliations:** 1 College of Oceanography and Ecological Science, Shanghai Ocean University, Shanghai 201306, China; wdzhu@shou.edu.cn (W.Z.); kfluan@shou.edu.cn (K.L.); 2 Shanghai Engineering Research Center of Estuarine and Oceanographic Mapping, Shanghai 201306, China

**Keywords:** ship instance segmentation, attention mechanism, convolutional neural network, vision transformer, remote sensing images

## Abstract

Ship instance segmentation in remote sensing images is essential for maritime applications such as intelligent surveillance and port management. However, this task remains challenging due to dense target distributions, large variations in ship scales and shapes, and limited high-quality datasets. The existing YOLOv8 framework mainly relies on convolutional neural networks and CIoU loss, which are less effective in modeling global–local interactions and producing accurate mask boundaries. To address these issues, we propose CDSANet, a novel one-stage ship instance segmentation network. CDSANet integrates convolutional operations, Vision Transformers, and attention mechanisms within a unified architecture. The backbone adopts a Convolutional Vision Transformer Attention (CVTA) module to enhance both local feature extraction and global context perception. The neck employs dynamic-weighted DOWConv to adaptively handle multi-scale ship instances, while SIoU loss improves localization accuracy and orientation robustness. Additionally, CBAM enhances the network’s focus on salient regions, and a MixUp-based augmentation strategy is used to improve model generalization. Extensive experiments on the proposed VLRSSD dataset demonstrate that CDSANet achieves state-of-the-art performance with a mask AP (50–95) of 75.9%, surpassing the YOLOv8 baseline by 1.8%.

## 1. Introduction

Instance segmentation, which combines object detection and semantic segmentation for pixel-level delineation of individual objects, is fundamental to computer vision applications such as autonomous driving and video surveillance [1]. In maritime scenarios using remote sensing imagery, instance segmentation enables accurate and efficient separation of individual ships from backgrounds, including ports, water surfaces, and coastal infrastructure. It also supports precise ship localization in both near-shore and offshore environments. This capability is essential for intelligent navigation systems, maritime traffic monitoring, and 3D maritime scene reconstruction, thereby significantly enhancing the safety and autonomy of modern maritime systems [2]. Compared with object detection, instance segmentation poses greater challenges, as it requires both precise object localization and pixel-level classification. Most existing approaches are mostly built on object detection frameworks.

Recent research on ship instance segmentation has explored multiple imaging modalities [3]. Among these, Synthetic Aperture Radar (SAR) imagery provides all-weather, day-and-night imaging capabilities and can penetrate clouds and haze, making it widely used in maritime surveillance. Public SAR datasets such as SSDD [4] and HRSID [5] have been established to support ship detection and segmentation tasks. However, SAR imagery is inherently affected by speckle noise, complex backscattering mechanisms, and sparse texture, which make fine-grained instance segmentation challenging. Consequently, various SAR-based approaches, including Context-Aware Net [6], LFG-Net [7], and the method proposed by Wang et al. [8], have been introduced to mitigate these modality-specific issues. In contrast, optical remote sensing imagery offers high spatial resolution, rich texture and color information, and spectral consistency with human vision [3,9], making it particularly suitable for precise instance-level segmentation. Nevertheless, optical sensors are susceptible to illumination variations and atmospheric interference, such as clouds, haze, and aerosols [10]. Furthermore, publicly available optical ship segmentation datasets remain limited. To address this gap, we developed a manually annotated visible light remote sensing dataset named VLRSSD, focusing on maritime scenes and specifically designed for ship instance segmentation tasks. This dataset enriches the training resources for deep learning models and promotes further research in maritime remote sensing applications.

Ship instance segmentation is crucial for precise localization and boundary delineation in maritime scenarios. However, this task remains challenging in the context of optical remote sensing due to several inherent factors. First, ships in nearshore port areas are often densely packed, making it difficult to separate individual instances. Second, ships in remote sensing images exhibit considerable variability. Differences in imaging altitudes can lead to large variations in ship sizes, while diverse ship categories—such as fishing boats, warships, and cargo vessels—display pronounced differences in shape and structure. The existing YOLOv8 framework primarily relies on convolution-based C2f modules, conventional convolutional blocks, and CIoU loss, which are limited in their ability to capture complex local–global interactions and generate precise mask boundaries.

To address these challenges, we propose a novel single-stage instance segmentation framework called CDSANet, which integrates convolutional neural networks (CNNs), Vision Transformers [10], attention mechanisms, and an enhanced loss function. CDSANet is built upon the YOLOv8 framework [11], which is known for its high accuracy and flexibility in both detection and segmentation tasks. Inspired by the lightweight design of MobileViT-v3 [12], we introduce the CVTA module, which leverages the efficiency of convolutions while retaining the global modeling capability of Transformers. This module overcomes the limited feature extraction capacity of the original C2f module when handling diverse ship types. Additionally, we design a new dynamic convolution module, DOWConv, and investigate its synergy with CVTA to enhance the model’s representational power and training efficiency. Together, these components improve ship instance segmentation performance in remote sensing images. CDSANet is primarily applicable to ship instance segmentation in nearshore ports and harbors under clear weather conditions, where ships are relatively densely packed compared to open-sea environments. The main contributions of this study are summarized as follows:

(1) A novel CVTA module that integrates CNN and Vision Transformer architectures to enhance global–local feature representation. Additionally, the CBAM [13] attention mechanism is incorporated into the backbone to further improve feature extraction.
(2) The network neck uses DOWConv, a dynamic convolution module that generates spatially and channel-wise adaptive kernels. This design better captures fine-grained features such as edges and shape variations while remaining computationally efficient.
(3) The loss function is upgraded to SIoU loss [14], enabling more precise instance localization and boundary refinement.
(4) We developed a novel dataset, VLRSSD, and employ a MixUp-based [15] image-level augmentation strategy to enhance segmentation performance.

The remainder of this paper is organized as follows. Section 2 reviews related works on instance segmentation, ship instance segmentation, and CNN–Transformer-based methods. Section 3 introduces the proposed CDSANet framework and describes the ship datasets employed in this study. Section 4 presents the experimental setup and results, while Section 5 discusses the limitations of the current work and provides further analysis. Finally, Section 6 concludes the paper and outlines directions for future research.

## 2. Related Work

### 2.1. Instance Segmentation

Current methods can be broadly categorized into two groups: two-stage and single-stage approaches.

Two-stage methods include Mask R-CNN [16], an extension of Faster R-CNN [17]. Mask R-CNN replaces the original RoIPooling layer with RoIAlign and incorporates a mask branch based on fully convolutional networks [18], enabling efficient instance segmentation. Cascade Mask R-CNN [18] builds upon the multi-stage bounding box regression framework of Cascade R-CNN by adding additional mask branches to further improve instance segmentation accuracy. CATNet [19] introduces three lightweight modules—DenseFPN, SCP, and HRoIE—which enhance instance segmentation performance in remote sensing images by aggregating contextual information at the feature, spatial, and instance levels.

Single-stage methods, inspired by anchor-free object detection approaches [20,21,22,23], streamline the pipeline by directly predicting an object’s position, category, and mask. SOLO [24] reformulates instance segmentation as a location prediction task using spatial encoding. SOLOv2 [25] is an optimized version of SOLO, incorporating further design improvements. YOLACT [26] generates final masks by producing shared prototype masks and combining them with instance weights from the detection branch. The instance segmentation branches of YOLOv5, YOLOv7 [27], and YOLOv8 [11] largely adopt the core concept of YOLACT, while implementing task-specific enhancements and optimizations to improve performance. These models further refine the approach through architectural modifications and targeted optimization strategies.

### 2.2. Ship Detection and Segmentation

Several recent studies have substantially improved segmentation and detection performance on SAR-based ship imagery. For example, Yasir et al. [28] propose a YOLOv7-based method for improved SAR ship instance segmentation. Shao et al. [29] introduce a scale-based SAR ship instance segmentation approach, constructing a multi-scale representation within a single scale. Wei et al. [7] present LFG-Net, a network that integrates low-level feature guidance with super-resolution denoising techniques specifically for SAR ship imagery. HTC+ [30] enhances segmentation accuracy on SAR images by combining seven specialized techniques. Lite-YOLOv5 [31] proposes a lightweight SAR ship detection model that achieves high detection accuracy while significantly reducing parameter size and computational cost. Wang et al. [32] introduce a multi-objective optimization method based on the novel firefly algorithm (NOFA) to optimize network architectures for SAR ship imagery. MLDet [33] is a multitask framework that combines angle-aware classification, speckle suppression, and Gaussian-mask segmentation to improve accuracy and robustness. SRNet [34] segments ships in SAR imagery using rotated bounding boxes and feature alignment to enhance accuracy in complex scenes. I-Polar Mask [35] improves near-shore ship segmentation through tailored polar representations and context-aware modules. DFRInst [36] dynamically refines features for improved ship instance segmentation on the high-resolution SISP dataset. GM R-CNN [9] leverages global semantic information with a Global Mask Head to achieve state-of-the-art results. DANet [37] introduces a dual-branch activation network with refined features and dual mask–activation branches to enhance small ship instance segmentation. Finally, MASSNet [3] improves the accuracy and efficiency of ship instance segmentation via a multi-scale attention mechanism.

### 2.3. Vision Transformers and CNN-ViT Hybrids

Since the introduction of the Transformer [38] architecture in NLP, researchers have extensively investigated its application in computer vision. ViT (Vision Transformer) [10] first demonstrated that modeling images as sequences of patches and applying a standard Transformer encoder could achieve state-of-the-art performance on large-scale classification benchmarks. To tackle the data inefficiency issue of ViT when trained on smaller datasets, DeiT [39] introduced distillation techniques, enabling ViT to achieve competitive results with less data and reduced training effort. To further improve scalability to dense prediction tasks, Swin Transformer [40] proposed a hierarchical architecture employing shifted-window self-attention, significantly reducing computational complexity while supporting detection and segmentation applications. Other ViT variants, such as T2T-ViT [41], PVT (Pyramid Vision Transformer) [42], and CvT (Convolutional Vision Transformer) [43], introduce progressive tokenization, pyramid structures, and convolutional embeddings to enhance local context modeling and efficiency. The STFF [44] and UTGLO [45] models employ self-supervised and unsupervised Transformers, respectively, to enhance semantic segmentation of SAR images by leveraging feature fusion and generative label optimization.

Since the introduction of the Transformer [38] architecture in NLP, researchers have extensively explored its applications in computer vision. ViT (Vision Transformer) [10] first demonstrated that modeling images as sequences of patches and applying a standard Transformer encoder can achieve state-of-the-art performance on large-scale classification benchmarks. To address the data inefficiency of ViT on smaller datasets, DeiT [39] introduces distillation techniques, enabling competitive results with less data and reduced training effort. To further improve scalability for dense prediction tasks, Swin Transformer [40] proposes a hierarchical architecture employing shifted-window self-attention, which significantly reduces computational complexity while supporting detection and segmentation applications. Other ViT variants, including T2T-ViT [41], PVT (Pyramid Vision Transformer) [42], and CvT (Convolutional Vision Transformer) [43], incorporate progressive tokenization, pyramid structures, and convolutional embeddings to enhance local context modeling and efficiency. Additionally, STFF [44] and UTGLO [45] employ self-supervised and unsupervised Transformer models, respectively, to enhance semantic segmentation of SAR images by leveraging feature fusion and generative label optimization.

CNNs efficiently capture local features with strong inductive biases but cannot model long-range dependencies, which are crucial in complex remote sensing scenarios, such as dense maritime environments. In contrast, ViTs excel at modeling global context but are computationally expensive. Combining CNNs and ViTs leverages their complementary strengths, improving both performance and efficiency. Several CNN–ViT hybrid models have demonstrated strong potential in complex visual tasks. DTNet [46] enhances camouflaged object segmentation by introducing boundary-aware and multi-level fusion modules that integrate CNN and ViT features. CMViT [47] incorporates a lightweight cross-module attention encoder to enable robust local–global feature interaction, supporting real-time detection on edge devices. MobileViT [48] and MobileViTv2 [49] integrate Transformer units into efficient CNN backbones, enabling lightweight global modeling while maintaining competitive performance in mobile-friendly vision tasks. MobileViTv3 [12] represents an efficient hybrid design, balancing accuracy and computational efficiency via lightweight convolutional blocks and ViT units. ViT-CoMer [50] is a pre-training-free ViT backbone that combines multi-receptive-field CNN features with bidirectional fusion, achieving strong instance segmentation performance.

Inspired by the hybrid local–global encoding pattern in MobileViTv3, we propose a lightweight CNN–ViT fusion module, termed CVTA, which employs split-path convolution and a simplified Transformer to enhance contextual feature modeling.

### 2.4. Dynamic Convolution and Attention Mechanisms

Dynamic convolution techniques demonstrate strong capability in capturing spatial and contextual variations by dynamically generating kernel weights conditioned on the input. Representative approaches include CondConv [51], Dynamic Convolution [52], and DOConv [33], each employing distinct mechanisms to achieve flexible and input-adaptive filtering. These methods have been widely adopted in various vision tasks to enhance feature representation. Building upon these developments, we design a lightweight and effective dynamic convolution module, termed DOWConv, which is described in Section 3.3. Additionally, we integrate attention modules, such as CBAM, into the backbone network to enhance spatial and channel-wise attention while maintaining low computational overhead.

For more precise localization, we adopt the SIoU loss, which incorporates shape and distance metrics into bounding box regression, thereby improving boundary refinement. By leveraging these established techniques, our model achieves enhanced segmentation accuracy and robustness on remote sensing ship images.

## 3. Method and Ship Data

### 3.1. Overall Structure

**Figure 1:**
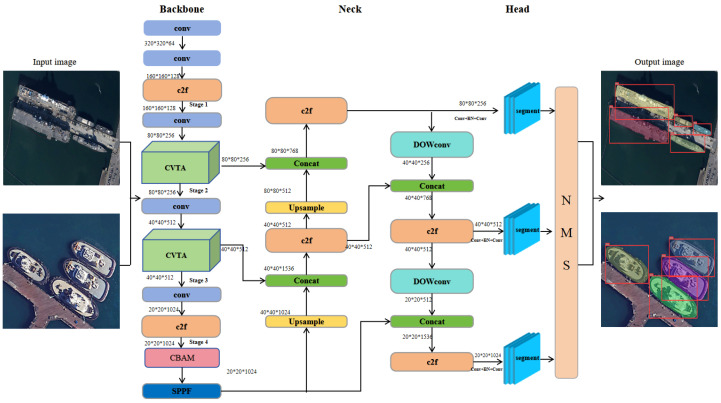
Overall architecture of the ship detection and segmentation model.

Figure 1 illustrates the structure of CDSANet, which consists of three main components: the backbone for feature extraction, the neck for feature refinement, and the detection heads for final predictions. The input image is first uniformly resized and normalized. The backbone extracts multi-scale features by integrating standard convolutional blocks, C2f modules, SPPF modules, CBAM, and the proposed CVTA module. This design allows the network to capture both rich semantic information and fine-grained details. The backbone produces hierarchical feature maps, which are then processed by the neck. Here, the standard convolution modules are replaced with the DOWConv modules to improve the model’s ability to capture fine-grained features, such as edges and shape variations. The neck outputs feature maps at three different scales, which are fed into their respective detection heads. Furthermore, the bounding box regression in the segmentation detection heads replaces the CIoU loss with the SIoU loss. In summary, the proposed framework achieves more accurate instance segmentation while maintaining computational efficiency and a compact model size. Detailed descriptions of the core components are provided in the following sections.

### 3.2. CVTA

**Figure 2:**
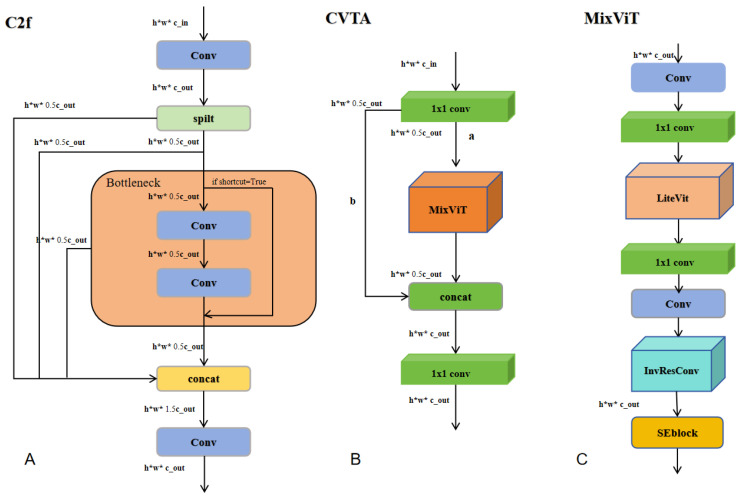
(**A**) baseline C2f module from YOLOv8; (**B**) proposed CVTA module integrating convolution and Transformer; (**C**) MixViT submodule for feature fusion.

To enhance the feature extraction capability of the C2f module (Figure 2A) in YOLOv8, which effectively captures local details but struggles with global contextual information in port and coastal scenes containing multiple ships of varying shapes, we introduce a novel hybrid module called CVTA (Figure 2B). Inspired by MobileViTv3 [12], which combines convolutional inductive biases with the global modeling capabilities of Transformers, CVTA employs a structured multi-branch design to explicitly partition and fuse features. In CVTA, convolution operations capture local spatial details, such as edges and contours, which are critical for ship targets, while Transformer units encode long-range contextual dependencies. Residual connections are strengthened to facilitate gradient propagation. This dual-path aggregation of local and global features improves feature robustness and instance awareness, particularly for densely packed or morphologically diverse ships. By explicitly modeling both local details and global context, CVTA overcomes the limitations of conventional C2f modules in capturing long-range dependencies while preserving fine-grained spatial information. This design enhances local–global feature interactions, producing richer and more discriminative representations for ship segmentation.

Inspired by the lightweight Vision Transformer architecture in MobileViTv3 [12] and the standard Transformer design [38], the proposed MixVit module integrates convolutional operations with a lightweight Transformer block, LiteVit, to balance local feature extraction and global context modeling. MixVit combines convolutional layers to preserve fine-grained textures and structural details of ships with LiteVit to capture long-range dependencies across the image. This design enables effective representation of both densely packed and morphologically diverse ships, enhancing segmentation robustness and accuracy.

LiteVit follows the efficient Transformer blocks used in MobileViTv3, incorporating multi-head self-attention and feed-forward layers with normalization to balance representational capacity and computational cost. MixVit further extends this design by integrating convolutional layers, such as InvResConv, with LiteVit, enabling more effective fusion of local and global features.

MixViT (Figure 2C) serves as the core component of CVTA, integrating standard convo lutional, pointwise (1 × 1) convolutional, and Vision Transformer–based mechanisms. It begins by performing channel transformation and fusion through a combination of standard and pointwise convolutional layers. Subsequently, additional convolutional operations and the LiteVit module are incorporated to strengthen feature learning and enhance training stability. At the tail of the CVTA module, the InvResConv block and SE attention mechanism are employed. The InvResConv block primarily utilizes pointwise and depthwise separable convolutions, while its multi-path feature fusion design enriches the representational diversity of extracted features. As a key feature extraction module in the backbone of CDSANet, the CVTA module with MixViT replaces several C2f modules to extract multi-scale and multi-level features. It provides more discriminative representations for subsequent attention modules and segmentation heads, thereby improving the overall performance and robustness of the model.

**Figure 3:**
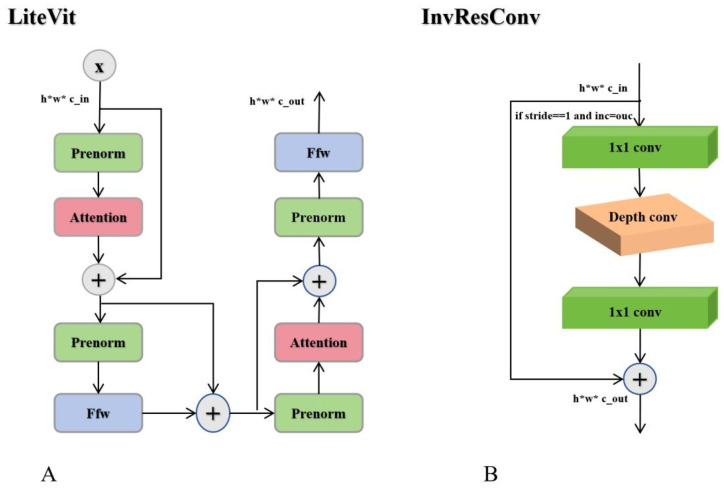
Structure of the CVTA components: (**A**) LiteViT submodule for global feature extraction; (**B**) InvResConv submodule for convolutional feature refinement. The “+” operation denotes residual connections that enhance gradient flow and feature reuse.

LiteVit (Figure 3A) is a lightweight Vision Tr Figure 3A ansformer encoder module designed to efficiently extract high-level semantic features for downstream tasks. It consists of two sequential layers, each composed of a pre-normalized multi-head self-attention mechanism and a pre-normalized feed-forward network. Layer normalization is applied before each operation to stabilize training, while residual connections preserve feature integrity and facilitate smooth gradient propagation. By modeling long-range dependencies and enabling expressive nonlinear transformations, LiteVit effectively enhances contextual feature representation.

InvResConv (Figure 3B) is inspired by the inverted residual block introduced in Mo bileNetV2 [53] and subsequently adopted in the MobileViT series, including MobileViTv3. This structure is particularly suitable for lightweight networks, as its expansion–projection bottleneck design and depthwise separable convolutions significantly reduce the additional computational overhead introduced by the Transformer. In our framework, InvResConv is integrated into the MixVit module to strengthen feature representation while maintaining computational efficiency. Compared to conventional residual blocks, InvResConv alleviates representational bottlenecks during channel expansion and reduction, making it more appropriate for resource-constrained scenarios such as real-time ship instance segmentation.

Specifically, the module first employs pointwise convolutions for channel expansion, followed by depthwise separable convolutions that independently process spatial information within each channel, thereby minimizing computational cost. A final pointwise convolution restores the original channel dimensions. When the stride equals 1 and the input and output channels are identical, a residual shortcut is applied.

**Figure 4:**
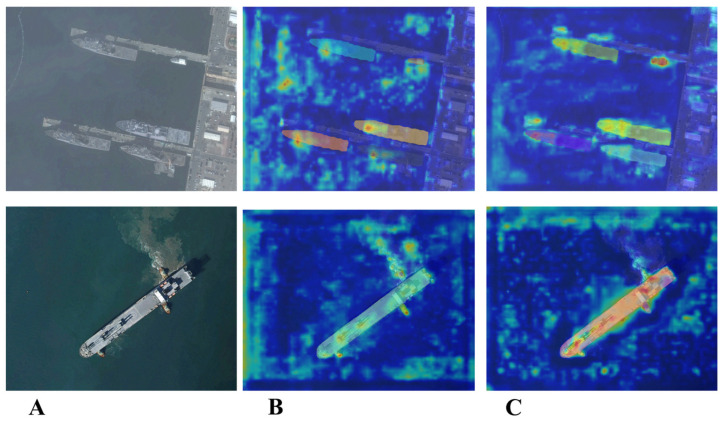
(**A**) is the original image, (**B**) is the heatmap after using C2f, (**C**) is the heatmap after using CVTA module.

To further illustrate the effectiveness of the proposed CVTA module, we present a visual comparison of heatmaps before and after replacing the original C2f with CVTA. As shown in Figure 4, CVTA not only suppresses background clutter but also more effectively captures ship regions and boundaries. These heatmaps are generated using the Grad-CAM [54] visualization method, where brighter areas indicate regions that contribute most strongly to the model’s prediction.

### 3.3. DOWConv

**Figure 5:**
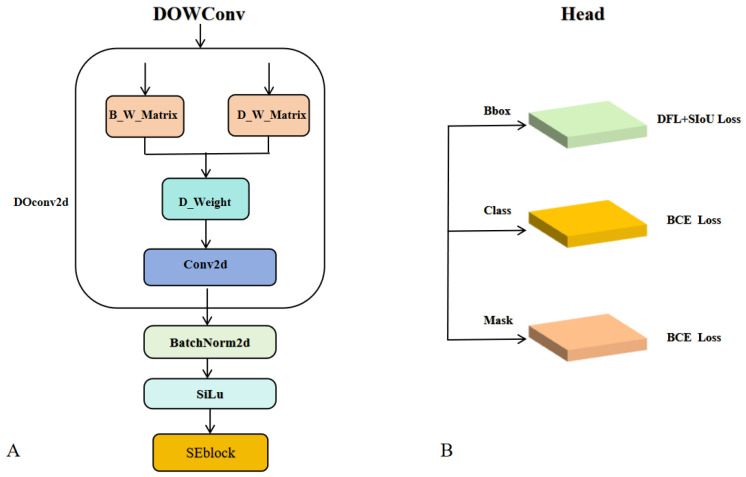
(**A**) DOWConv module for dynamic convolutional feature aggregation (**B**) is the module head, where the SIoU loss function is used for Bbox.

Inspired by DOConv [55], we propose a novel convolutional module called Dynamic Orthogonal Weight Convolution (DOWConv), which replaces two standard convolutional layers in the neck to enhance feature representation. Traditional convolutional layers rely on static kernels that struggle to adapt to objects with varying shapes, scales, and orientations. To address this limitation, DOWConv dynamically generates spatially adaptive kernels that can better capture fine-grained structural features while maintaining high computational efficiency. As illustrated in Figure 5A, the DOWConv module consists of a DOConv2d layer, batch normalization, an activation function, and a squeeze-and-excitation (SE) block. The DOConv2d layer includes two learnable weight matrices: (1) a base weight matrix (B_W_Matrix), initialized using Kaiming initialization to provide the kernel’s foundational parameters; and (2) a dynamic weight matrix (D_W_Matrix), which incorporates a diagonal matrix D_diag_ to ensure stable and adaptive behavior during initialization.

Then, the interaction between the base and dynamic weight matrices produces the final dynamic weights (D_Weight) used in the convolution operation, followed by a standard convolution computation. This process can be summarized in three steps: (1) generating the initial base weights; (2) constructing the dynamic weights modulated by D_diag_; and (3) combining both to compute the spatially adaptive kernels. Unlike traditional convolutional layers with fixed kernel weights, DOWConv generates dynamic, spatially and channel-wise adaptive kernels, enabling the network to more effectively capture fine-grained features such as object edges, shape variations, and scale changes. This dynamic mechanism allows each convolutional kernel to adapt to different spatial locations and feature channels, thereby improving the network’s sensitivity to subtle differences in ship shapes, sizes, and orientations.

### 3.4. SIoU and Attention Mechanism

We integrate the SIoU loss and attention mechanisms to enable the model to more effectively capture subtle shape variations, precise boundaries, and spatial relationships. Specifically, this study replaces the standard Complete IoU (CIoU) loss with the Scalable IoU (SIoU) loss function for bounding box regression. SIoU extends the conventional Intersection-over-Union metric by incorporating additional geometric properties of the bounding box, such as the center distance, orientation angle, and aspect ratio. This joint optimization of spatial alignment and shape consistency enhances the model’s ability to distinguish densely packed ships and improves localization accuracy as well as boundary quality in instance segmentation tasks. The mathematical formulation of the SIoU loss function is defined as

(1) $$L_{SIoU}=1-IoU+\frac{∆+\theta }{2}$$

where IoU denotes the standard Intersection-over-Union ratio, and ∆ is a distance-based term that measures the Euclidean distance between the centers of the predicted and ground-truth bounding boxes. It also accounts for the relative orientation of this center offset with respect to the box dimensions, assigning larger penalties when the predicted box center deviates more along the object’s principal axes. This encourages more accurate localization by penalizing misalignment in both the horizontal and vertical directions. Moreover, θ is a shape-based term that quantifies the differences in width and height between the predicted and ground-truth boxes. It increases the loss when the predicted box’s scale or aspect ratio diverges from the ground truth, thereby improving the model’s robustness to objects with diverse sizes and shapes. This component ensures that both large and small, as well as wide and narrow objects, are accurately represented.

Meanwhile, the CBAM and SE attention modules dynamically emphasize distinctive ship features while suppressing irrelevant background information, thereby enhancing segmentation robustness across diverse ship morphologies. CBAM sequentially applies channel and spatial attention mechanisms, enabling adaptive focus on salient regions while attenuating uninformative features. It is particularly effective in capturing fine-grained object boundaries and positional cues. Based on extensive empirical evaluations, CBAM is applied three times before the SPPF module to enhance spatially aware feature refinement and further improve segmentation accuracy.

**Figure 6:**
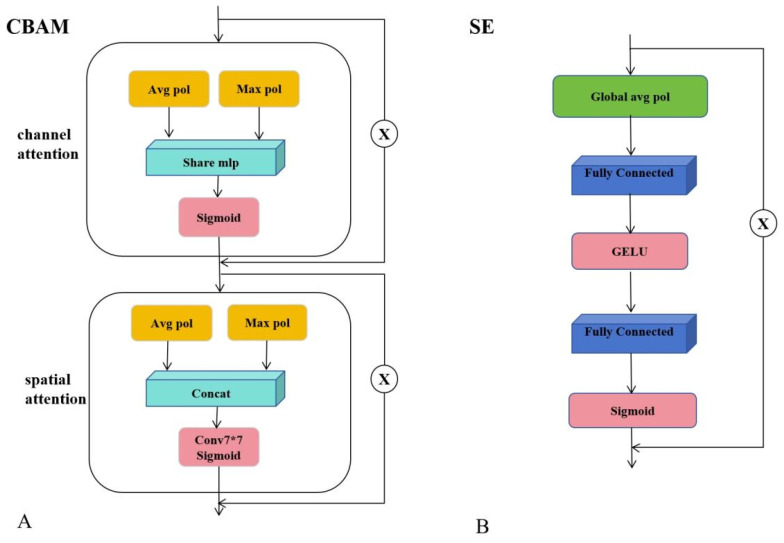
(**A**) CBAM module for channel and spatial attention; (**B**) SE module for channel-wise feature recalibration. The symbol “×” denotes element-wise multiplication between the input and the output of the attention submodule.

In contrast, the SE [56] module performs channel-wise feature recalibration and exhibits strong stability and generalization, particularly when integrated into lightweight architectures such as MobileViT. Consequently, SE blocks are embedded within both the CVTA and DOWConv modules to strengthen their representational capacity without introducing significant computational overhead. The overall network architecture is illustrated in Figure 6.

### 3.5. Ship Data

#### 3.5.1. VLRSSD Dataset

Due to potential geopolitical and military sensitivities, publicly available visible light remote sensing ship datasets for instance segmentation remain scarce. To address this limitation, we constructed and manually annotated a new dataset termed VLRSSD (Visible Light Remote Sensing Ship Dataset). The annotation format and labeling scheme follow the MS COCO standard, supporting polygon-based instance segmentation.

The dataset comprises 2600 high-resolution visible light images of ships collected from a wide range of geographical regions and acquisition sources. Specifically, 1047 images were obtained from the HRSC2016 dataset, while the remaining 1553 images were manually extracted from Google Earth imagery covering major international ports across Asia, Europe, and North America. The selected ports and adjacent offshore areas include Shanghai, Zhoushan, Tianjin, Qingdao, Shenzhen, Hong Kong, Singapore, Dubai, Pearl Harbor, Busan, Tokyo, Kaohsiung, Amsterdam, and San Francisco, ensuring diverse maritime and geographical representations. The VLRSSD dataset incorporates imagery captured by multiple satellite sensors, including both publicly available and high-resolution commercial satellites, leading to natural variations in spatial resolution and imaging perspectives. Most images were collected under clear-sky conditions, while a smaller subset includes cloudy, foggy, or overcast scenes, further enhancing atmospheric diversity. In total, 13, 12, and 156 images correspond to cloudy, foggy, and open-sea conditions, respectively. The majority of images depict coastal and port regions, with a smaller subset representing offshore vessels in open waters.

**Figure 7:**
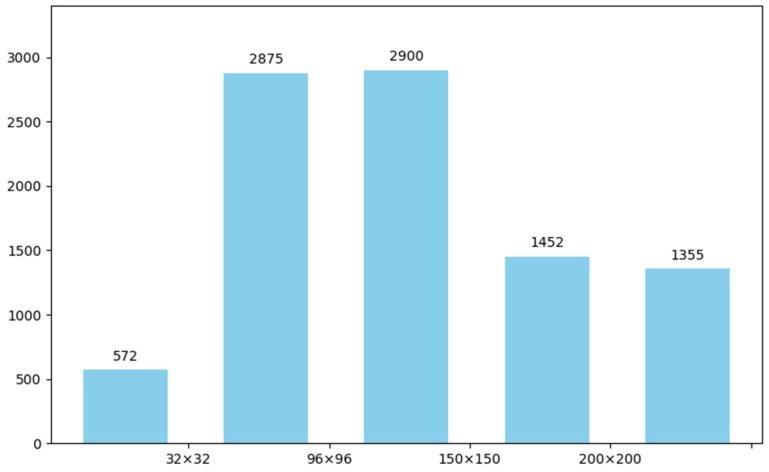
Distribution of ship instance sizes.

**Figure 8:**
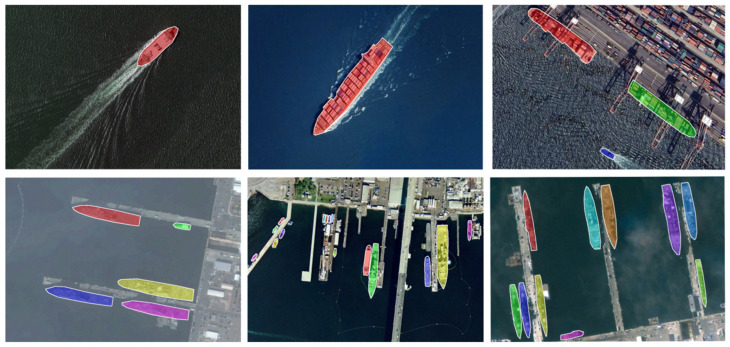
Examples from VLRSSD dataset with annotations.

The dataset is divided into training and validation subsets with an 8:2 ratio, containing 2080 images for training and 520 for validation. A total of 9154 ship instances are annotated with fine-grained polygonal masks, averaging 3.52 ships per image. To enhance generalization across diverse maritime environments, all ships are labeled as a single category without distinguishing between vessel types. Summary statistics of the dataset are provided in Table 1, and the distribution of ship instance sizes, measured by pixel area, is illustrated in Figure 7, where the x-axis represents instance area and the y-axis indicates the number of ships. Representative annotation examples are shown in Figure 8.

All images in the VLRSSD dataset were manually annotated using LabelMe and subsequently reviewed by at least two annotators. Polygon masks were refined with particular care in regions featuring complex backgrounds, such as wakes, piers, and coastal structures. Common sources of annotation uncertainty include wakes occasionally misidentified as part of the ship, clutter from nearby piers or buildings, partially visible vessels, and small or irregularly shaped ships. These challenges may lead to segmentation errors or reduced precision in distinguishing ships from visually similar surroundings. We explicitly report these potential issues to assist readers in interpreting model performance.

#### 3.5.2. MariShipInsSeg Dataset

The MarShipInsSeg dataset [9] consists of optical images annotated in the COCO format. Most images were collected from web sources, supplemented by ship samples from the Sea Ship, COCO, and VOC datasets. It encompasses a wide range of ship categories, including cargo ships, cruise liners, warships, fishing vessels, and coastal ships. In total, the dataset contains 4001 images and 8413 annotated ship instances, with an average of approximately 2.1 ships per image. Although MarShipInsSeg is an optical (visible light) ship dataset rather than a remote sensing dataset, it was adopted in this study due to the limited availability of public optical remote sensing ship datasets. Moreover, its texture and color characteristics are highly consistent with those of optical remote sensing imagery. Therefore, it serves as a suitable benchmark to further evaluate the generalization and robustness of the proposed model. This evaluation also indirectly demonstrates that the model maintains strong performance on standard optical images. As an optical image dataset designed for visual scene analysis, MarShipInsSeg provides high-quality imagery with diverse ship categories, varying instance densities, and broad size distributions. Additional details are summarized in Table 1.

### 3.6. Improving Generalization via MixUp

To mitigate the limitation of insufficient training data, and enhance the generalization capability of our network, we adopt MixUp [15], an image-level data augmentation strategy which generates new samples by linearly interpolating pairs of existing training samples. This method introduces additional variations in the training distribution while preserving structural coherence, which is crucial for instance segmentation.

Given two randomly selected samples ${(x}_{i},y_{i})$ and ${(x}_{j},y_{j})$ from the training set (typically within the same batch), MixUp generates a synthetic training sample by linearly combining their images and labels. Specifically, the new synthetic sample $\left(x_{k},y_{k}\right)$ is obtained as

(2) $$x_{k}=\lambda x_{i}+(1-\lambda )x_{j},y_{k}=\lambda y_{i}+\left(1-\lambda \right)y_{j},$$

where x denotes the image and y represents the corresponding label. The mixing coefficient λ is sampled from a Beta distribution:

(3) λ~Beta(α,α),λ∈[0,1].

Here, α represents the shape parameter of the Beta distribution, which controls the degree of interpolation between two samples. In our implementation, α is set to 32.0, resulting in a λ distribution centered around 0.5. Empirical observations indicate that MixUp augmentation is particularly effective in enhancing segmentation performance for small ships. By moderately blending image–label pairs, MixUp produces diverse yet structurally consistent training samples, thereby mitigating overfitting in cluttered maritime scenes and exposing the network to richer contextual variations while preserving object-level semantics. Previous studies on MixUp [15] and CutMix [57] have similarly demonstrated that moderate sample blending improves dense prediction tasks by maintaining structural integrity and enhancing model generalization.

## 4. Experiment and Result

### 4.1. Experimental Setup and Experimental Details

All experiments were conducted on a Windows 11 operating system. The hardware setup included a 13th Gen Intel(R) Core (TM)i9-13900KF CPU and an NVIDIA GeForce RTX 4090 GPU with 56 GB of dedicated memory. The software environment was configured with CUDA 12.6 and PyTorch 2.0.0. For all ablation experiments, the models were trained from scratch without pre-trained weights to ensure a fair comparison focused solely on architectural improvements. The training hyperparameters were as follows: an initial learning rate of 0.003, a final learning rate of 0.1, a random seed of 0, both num_workers and batch size set to **8**, and a total of 150 epochs. All other training parameters followed the default configuration of YOLOv8. All input images were uniformly resized to 640 × 640 pixels. For comparative experiments involving other network architectures, training was performed using MMDetection 3.3 [58],with each model trained for 30 epochs. Owing to MMDetection’s efficient training pipeline and rapid convergence, extending the training duration often led to overfitting. Empirical observations showed that 30 epochs were sufficient to achieve optimal performance. All other training settings adhered to the default MMDetection configuration.

### 4.2. Evaluation Metrics

To provide a quantitative and comprehensive assessment of instance segmentation performance, this paper utilizes the Average Precision (AP) [59] metric. Given that AP is based on precision and recall, we first introduce these two fundamental metrics to facilitate a better understanding of the AP calculation. Precision is defined as the proportion of correctly predicted instances to the total number of predicted instances, as shown in Equation (4):

(4) $$Precision=\frac{TP}{TP+FP}$$

Here, TP denotes true positives and FP denotes false positives. Recall is the proportion of correctly identified instances among all actual positive samples, as shown in Equation (5):

(5) $$Recall=\frac{TP}{TP+FN}$$

Here, FN denotes false negatives. In instance segmentation, Average Precision (AP) evaluates both the accuracy of object localization and the quality of predicted masks. The COCO-style AP, computed as the average over multiple IoU thresholds ranging from 0.5 to 0.95 (denoted as AP_0.5:0.95_, hereafter abbreviated as AP), provides a comprehensive measure of the model’s performance under both loose and strict matching conditions. Specifically, AP_50_ (IoU ≥ 0.5) indicates the model’s ability to roughly localize objects, while AP_75_ (IoU ≥ 0.75) evaluates its precision in capturing mask boundaries. By averaging AP across a range of IoU thresholds, this metric offers a balanced evaluation, penalizing models that perform well under loose matching but poorly at stricter thresholds. AP can also be reported for objects of different sizes—small (AP_S_), medium (AP_M_), and large (AP_L_)—providing detailed insights into performance across varying scales, such as small or densely packed ships. Mathematically, AP is calculated as the area under the precision–recall curve, representing the definite integral of recall with respect to precision, as shown in Equation (6):

(6) $$AP=\int_{0}^{1}P\left(R\right)d(R)$$

Here, *P*(*R*) denotes precision as a function of recall. In this study, multiple evaluation metrics, including AP, AP_50_, AP_75_, AP_S_, AP_M_, and AP_L_, are employed to comprehensively assess the model’s segmentation performance, ensuring that both localization accuracy and mask quality are thoroughly evaluated.

### 4.3. Ablation Study

#### 4.3.1. Effect of Key Modules and Augmentation Strategies

Table 2 presents the results of ablation experiments conducted on the VLRSSD dataset. All experiments were conducted using 640 × 640 pixel input images using the original model architecture. The baseline model achieved an AP of 74.1%, with AP_50_ at 94.4% and AP_75_ at 87.0%. In the first ablation experiment, two CVTA modules were added to the backbone, increasing AP to 74.5% and AP_50_ to 95.1%, although AP_75_ and AP_M_ exhibited minor decreases. In the second experiment, CVTA and DOWConv were incorporated into the baseline, yielding an AP of 74.8%, AP_50_ of 94.8%, and AP_75_ of 87.1%. In the third experiment, the integration of CVTA, SIoU, and CBAM resulted in an AP of 74.6%, with AP_50_ and AP_75_ reaching 94.7% and 87.1%, respectively. In the fourth experiment, integrating CVTA, DOWConv, SIoU, and CBAM achieved better overall performance, with AP rising to 75.2%, AP_75_ to 87.9%, and AP_L_ reaching 82.7%. In the fifth experiment, only the MixUp data augmentation strategy was applied. The results demonstrate its notable effectiveness for small-object detection, achieving an AP_S_ of 26.1%. Finally, combining all proposed components—CVTA, DOWConv, SIoU, CBAM—with MixUp produced the best overall results. The model achieved an AP of 75.9%, AP_50_ of 95.8%, AP_75_ of 89.8%, AP_S_ of 28.0%, AP_M_ of 65.2%, and AP_L_ of 83.1%.

To further evaluate the generalization capability of the model, we conducted ablation experiments on the public MariShipInsSeg dataset. Table 3 shows consistent performance improvements with the addition of each key module, and the full model achieves the highest AP of 49.3%, outperforming the baseline by 2.7%. The model also shows robust performance across various object sizes.

Table 2 presents the results of ablation experiments conducted on the VLRSSD dataset. All experiments used 640 × 640 pixel input images with the original model architecture. The baseline model achieved an AP of 74.1%, with AP_50_ of 94.4% and AP_75_ of 87.0%. In the first ablation, two CVTA modules were added to the backbone, increasing AP to 74.5% and AP_50_ to 95.1%, although AP75 and APM showed minor decreases. The second experiment incorporated both CVTA and DOWConv into the baseline, yielding an AP of 74.8%, AP50 of 94.8%, and AP75 of 87.1%. In the third experiment, the combination of CVTA, SIoU, and CBAM resulted in an AP of 74.6%, with AP_50_ and AP_75_ reaching 94.7% and 87.1%, respectively. The fourth experiment, which integrated CVTA, DOWConv, SIoU, and CBAM, improved overall performance, with AP rising to 75.2%, AP_75_ to 87.9%, and APL to 82.7%. In the fifth experiment, only the MixUp data augmentation strategy was applied, demonstrating its effectiveness for small-object detection with an AP_S_ of 26.1%. Finally, combining all proposed components—CVTA, DOWConv, SIoU, CBAM, and MixUp—produced the best overall results: AP of 75.9%, AP_50_ of 95.8%, AP_75_ of 89.8%, AP_S_ of 28.0%, AP_M_ of 65.2%, and AP_L_ of 83.1%.

To further evaluate generalization, ablation experiments were conducted on the public MariShipInsSeg dataset. As shown in Table 3, the addition of each key module consistently improved performance, with the full model achieving the highest AP of 49.3%, surpassing the baseline by 2.7%. The model also exhibited robust performance across objects of varying sizes.

**Figure 9:**
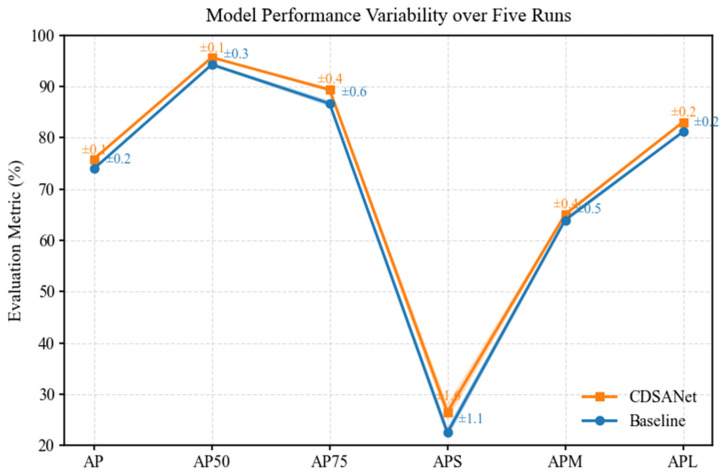
Model performance across five independent runs. Error bars indicate the standard deviation, highlighting the stability and consistency of the proposed method.

All reported results are based on single-run experiments. To assess stability and robustness, we additionally performed five independent runs on the VLRSSD dataset, achieving an average AP of 75.9. The baseline model was also trained five times under the same settings. The baseline model was also trained five times under the same settings, and the variance curves for both the baseline and proposed models are shown in Figure 9.

#### 4.3.2. Effect of MixUp Hyperparameter and CVTA Module Replacement

We first conducted an ablation study on the MixUp hyperparameter α to assess its impact on model performance. As reported in the original MixUp paper, there is no theoretically optimal value for α; instead, the authors empirically found that values between 0.2 and 0.4 produced strong results on the large-scale ImageNet classification dataset. This suggests that moderate interpolation between samples can enhance generalization while preserving original image content when sufficient training data is available. In our case, however, the dataset is relatively small, and the task focuses on instance segmentation, which typically benefits from stronger regularization. Under these conditions, larger α values can enforce stronger sample interpolation, increasing data diversity and improving feature generalization. To investigate this effect, we systematically tested multiple α values (0.3, 8, 16, 24, 32, and 36). As summarized in Table 4, α = 32 yielded the best overall performance, consistent with our expectations. These results demonstrate that the choice of α significantly affects final accuracy, and larger values are more suitable for our specific experimental setting.

In addition, we conducted an ablation study on the number of CVTA modules to investigate their impact on model performance. Specifically, we progressively replaced the C2f modules in different backbone stages with CVTA modules. As shown in Table 5 (where 1–4 indicate the positions of the C2f modules in the backbone, as illustrated in Figure 1), replacing the C2f modules in stages 2 and 3 yielded the best performance. This is because these intermediate stages balance spatial resolution and semantic abstraction, allowing the CVTA modules to effectively capture long-range dependencies while retaining sufficient structural details of ships. In contrast, replacing modules in the first stage led to degraded performance, as this stage primarily processes low-level features such as edges and textures, and introducing CVTA here can over-complicate the representation and hinder precise local feature extraction. Similarly, replacing modules in the fourth (deepest) stage was suboptimal, since this stage contains highly abstract semantic features, and adding CVTA can over-emphasize global dependencies at the expense of fine-grained ship structures. These findings highlight the rationale behind CVTA module placement: early-stage replacements compromise low-level feature extraction, while deeper replacements tend to over-abstract high-level semantics, both leading to degraded results. Moreover, when additional modules were replaced in the neck, training could not be completed due to GPU memory limitations (denoted as “OOM” in Table 5). Therefore, we adopted the configuration of replacing the C2f modules in stages 2 and 3, which achieved the highest accuracy under the current experimental conditions. These results provide empirical support for the placement of CVTA modules, demonstrating that their effectiveness depends on selecting stages that balance low-level and high-level feature representations.

### 4.4. Main Result

#### 4.4.1. Comparison with Other Models

**Figure 10:**
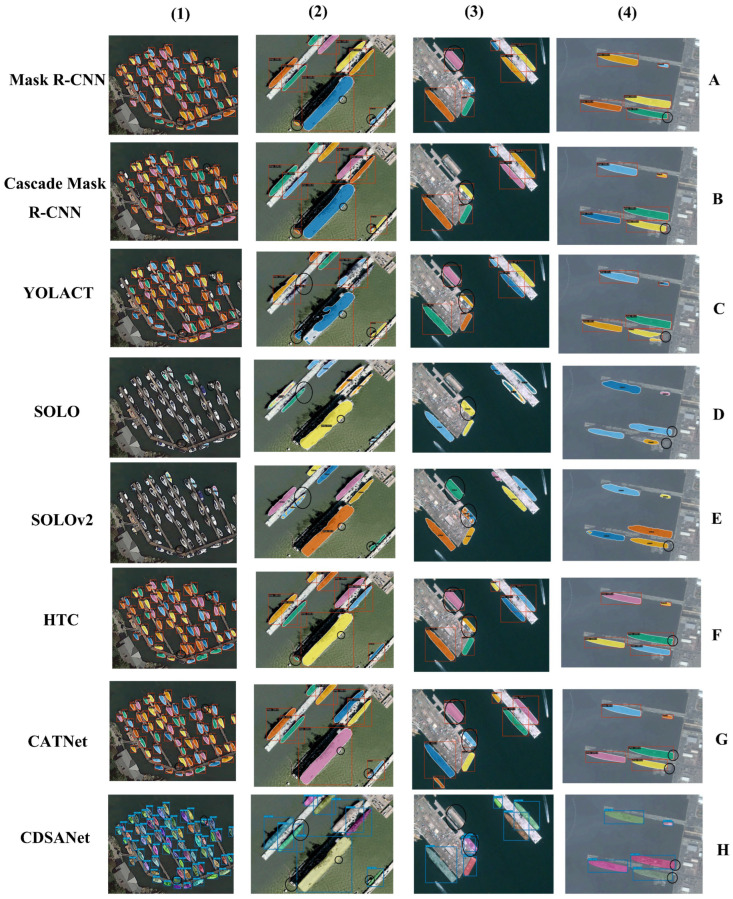
Instance segmentation results on the DOTA and VLRSSD (**A**) Mask R-CNN; (**B**) Cascade Mask R-CNN; (**C**) YOLACT; (**D**) SOLO; (**E**) SOLOv2; (**F**) HTC; (**G**) CATNet; (**H**) CDSANet.

Figure 10 presents qualitative results of our model compared with state-of-the-art algorithms on the VLRSSD and DOTA datasets, further validating the effectiveness of CDSANet for ship instance segmentation. Rows 1 to 8 show the segmentation outputs of Mask R-CNN [16], Cascade Mask R-CNN [18], YOLACT [26], SOLO [24], SOLOv2 [25], HTC [60], CATNet [19], and CDSANet, respectively. Compared to these methods, CDSANet accurately identifies and separates ship instances across various scenarios, reducing false positives and missed detections. As shown in Figure 10, other algorithms often suffer from misclassification, missed detections, incomplete segmentation of edge details, and limited performance in detecting small targets. In contrast, CDSANet generates masks that closely match the ground truth, particularly in terms of segmentation details, small object detection, and separation of overlapping ships, demonstrating superior mask completeness. In the first column, CDSANet achieves more complete segmentation of ships in densely populated port areas, successfully identifying some small vessels. In the second column, other models either fail to recognize a small ship located centrally, merge it with another nearby vessel, or misidentify ground objects as ships. The third column shows that other models produce incomplete edge segmentation and occasionally misclassify objects in the lower-left and lower-right corners as ships. In the fourth column, CDSANet outperforms other models in both mask boundary details and overall segmentation accuracy. Overall, only CDSANet consistently achieves completeness in ship identification, small ship detection, and edge segmentation.

As shown in Table 6 and Table 7, we compared CDSANet with several state-of-the-art instance segmentation methods on the VLRSSD and MariShipInsSeg datasets. CDSANet consistently outperforms all other models across various evaluation metrics, demonstrating its superiority in ship instance segmentation, particularly for small targets.

In Table 8, we report CDSANet’s performance on boundary-sensitive metrics (Boundary-AP and F-measure on a narrow band) as well as aspect ratio- (Tall/Medium/Wide) and instance density-based (Sparse/Medium/Dense) AP. These results further validate the effectiveness of CDSANet in segmenting ships of various shapes and densities. In Table 9, we evaluate the computational efficiency and model compactness of CDSANet. Although it does not surpass the lightweight baseline in terms of parameter count, model size, or frames per second (FPS), CDSANet still demonstrates significant advantages in segmentation accuracy over existing state-of-the-art instance segmentation methods. Specifically, on an NVIDIA RTX 4090 GPU, it achieves a model size of 48.7 MB, 25.3 million parameters, 111.4 GFLOPs, and 130.3 FPS, while we also report FPS and latency (ms) on a resource-constrained laptop equipped with an RTX 4070 GPU.

#### 4.4.2. Precision–Recall Curve Analysis

**Figure 11:**
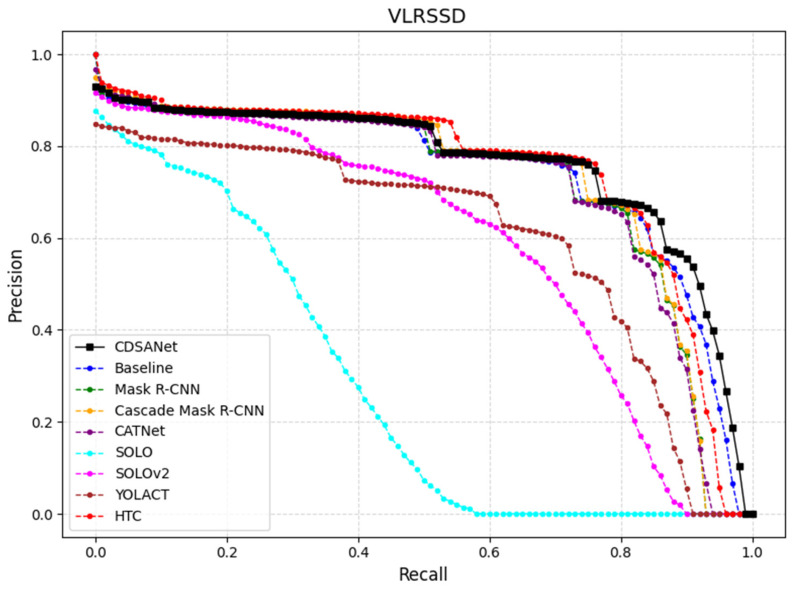
PR curves of different models on the VLRSSD dataset.

Figure 11 shows the average precision–recall (PR) curves of our method and other mainstream models evaluated on the VLRSSD dataset (IoU = 0.5 to 0.95). It can be seen that our proposed model (black curve) outperforms the others overall, with the PR curve covering the largest area, indicating superior average precision (AP). In the high-precision region, our model effectively reduces false positives, enhancing prediction reliability. In the high-recall region, it minimizes missed detections, achieving an optimal balance between precision and recall. Overall, CDSANet demonstrates superior performance in terms of precision, recall, and robustness, further validating its effectiveness for ship instance segmentation on the VLRSSD dataset.

#### 4.4.3. Error Analysis

To gain a more comprehensive understanding of CDSANet’s performance, we conducted an in-depth analysis using the COCO error analysis tool on the VLRSSD dataset. Table 10 presents the error analysis results for the Baseline, CATNet, HTC, and CDSANet models. C_50_ and C_75_ denote the average precision (AP) at IoU thresholds of 0.50 and 0.75, respectively. Loc refers to localization errors, reflecting the model’s inability to accurately locate objects. Sim denotes misclassification into similar categories within the same super-category, while Oth accounts for confusions with dissimilar object classes. BG indicates false positives in background regions, and FN represents false negatives, i.e., ground-truth instances that the model fails to detect.

On the VLRSSD dataset, CDSANet outperforms all competing methods, achieving C_50_ and C_75_ scores of 95.8% and 89.8%, respectively—surpassing HTC by 2.4% and 3.2%, and the Baseline by 1.4% and 2.8%. The model also achieves the lowest localization error of 1.0%, indicating highly precise instance positioning, and maintains the lowest false negative rate (1.0%), tied with the Baseline. Although the background false positive rate slightly increases to 2.2% compared to some advanced models, it remains lower than that of the Baseline. This minor increase is likely due to the complexity of maritime scenes; nevertheless, CDSANet demonstrates strong capability in minimizing missed detections and accurately delineating instance boundaries.

**Figure 12:**
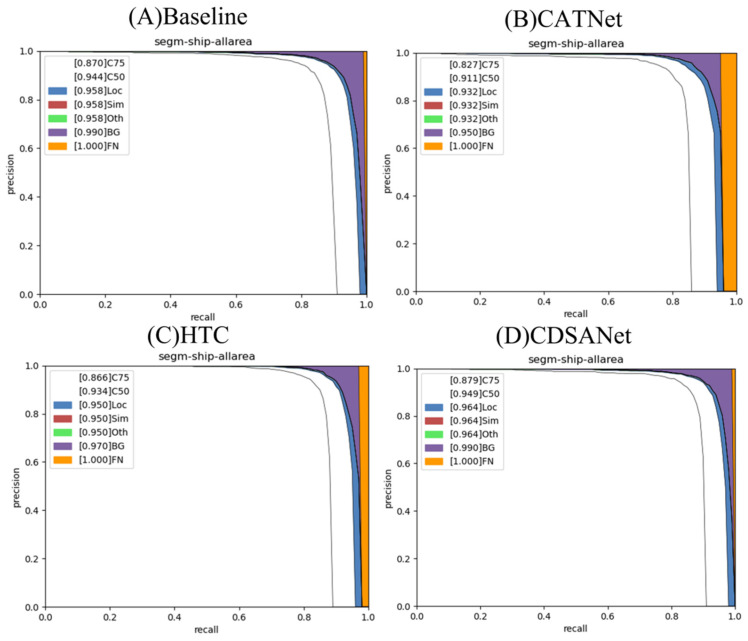
Error analysis results (**A**) Baseline (**B**) CATNet (**C**) HTC (**D**) CDSANet.

Overall, CDSANet achieves very high precision and excellent localization on the VLRSSD dataset, with significant gains in C_50_ and C_75_ scores. It attains the lowest miss rate and localization error, demonstrating accurate detection and precise positioning. Although its background false positive rate is not the lowest among all models, the overall error remains low, without compromising model stability. Figure 12 visually presents the error comparisons from Table 10, highlighting CDSANet’s advantages over the Baseline, HTC, and CATNet.

## 5. Limitations and Discussion

The VLRSSD dataset was collected from Google Earth, which inherently sources imagery from multiple sensors. Consequently, it can be considered a multi-source remote sensing dataset. However, Google Earth does not provide explicit annotations linking images or regions to specific sensor sources, making precise categorization by sensor type impossible. Additionally, most images depict nearshore ships under clear-sky conditions, while scenarios such as cloudy, overcast, or foggy weather, open-sea environments, and scenes containing ship wakes or waves are relatively scarce. These characteristics indicate limited scene diversity; strictly speaking, the dataset does not fully represent complex maritime environments. Furthermore, the high cost of annotation constrains the dataset size, which in turn limits the model’s generalization ability across diverse scenarios.

Due to the scarcity of ship datasets in complex maritime environments, further validation is needed to assess CDSANet’s performance in such challenging scenarios. Compared with widely adopted frameworks such as Mask R-CNN and Cascade Mask R-CNN, CDSANet converges more slowly and requires additional training epochs to reach optimal accuracy. As shown in Table 9, CDSANet achieves a favorable balance in model size, parameter count, FLOPs, and inference speed compared with state-of-the-art segmentation models. Nevertheless, it still lags behind the lightweight YOLOv8 baseline. Additionally, CDSANet is currently implemented on an early version of YOLOv8 and may not fully exploit the architectural and optimization improvements introduced in later versions such as YOLOv11. Although YOLOv11 offers certain advantages, it still primarily relies on conventional CNN backbones and CIoU-based loss functions, which limit global feature modeling and boundary localization. On our dataset, YOLOv11, three versions ahead of YOLOv8, achieves only modest performance improvements.

## 6. Conclusions and Future Work

In this study, we propose CDSANet, a novel ship instance segmentation model that integrates CNNs, vision transformers, dynamic convolution, attention mechanisms, the SIoU loss function, and a MixUp-based data augmentation strategy. CDSANet effectively addresses several limitations of YOLOv8, including its relatively weak capabilities in global context modeling, local–global feature interaction, feature extraction, and precise mask localization. As a result, CDSANet achieves superior segmentation accuracy and mask integrity, particularly for dense ship targets and objects with significant shape variations in optical remote sensing images, mainly in nearshore port and harbor areas under clear weather conditions. In addition, this work contributes a visible light remote sensing ship image dataset, providing a valuable resource for advancing research on nearshore ship instance segmentation. Despite the promising performance of the proposed model, its applicability is constrained by the limited diversity of available datasets. Future work could focus on enhancing domain generalization through domain adaptation techniques to mitigate performance degradation when transferring across different sensors or imaging environments. Moreover, integrating multimodal data, such as SAR and optical imagery, may provide complementary spatial–spectral cues, further improving segmentation accuracy and robustness.

Looking ahead, emerging architectures such as Mamba networks are attracting increasing attention compared to Transformers due to their efficiency in modeling long sequences, lower memory usage, and reduced computational costs. Therefore, future research will explore integrating Mamba [65] networks with more advanced YOLO series models to further improve segmentation efficiency and accuracy. Meanwhile, we recognize the robustness and efficiency of the MMDetection framework in instance segmentation tasks. Accordingly, we plan to strengthen research on model compression and efficient architectures within this framework, while also investigating how to incorporate the innovative concepts of Mamba networks into MMDetection.

It should be noted that achieving accurate ship segmentation in complex maritime environments remains a highly challenging task. The term “complex maritime environments” encompasses a wide range of conditions, and developing a comprehensive algorithm capable of handling all such scenarios is undoubtedly difficult. Therefore, future work will focus on a representative scenario within complex maritime conditions: ship segmentation under foggy and hazy weather. This scenario is both scientifically interesting and practically important, as fog and haze frequently occur at sea and can significantly impact maritime traffic and the safety of maritime trade. Fortunately, mature fog and haze scene image simulation methods can help alleviate the scarcity of such images under real-world conditions. We believe that combining the advantages of Mamba networks with ship instance segmentation in foggy and hazy remote sensing images can open new research opportunities and yield promising results.

## Figures and Tables

**Table 1:** Detailed ship imagery data.

Attribute	VLRSSD	MariShipInsSeg
Image number	2600	4001
Number of instances	9154	8413
Source	HRSC2016/Google Earth	internet/sea ship/coco/voc
Image size	300 × 300–1500 × 900	/
Type	Visible light remote sensing	Visual image
Task	Instance segmentation	Instance segmentation
Atmospheric conditions	clear, cloudy, foggy, overcast	/

**Table 2:** Ablation study on the VLRSSD dataset.

CVTA	DOWconv	SIoU	CBAM	Mixup	AP (%)	AP_50_ (%)	AP_75_ (%)	AP_S_ (%)	AP_M_ (%)	AP_L_ (%)	GFLOPS	Para (M)
-	-	-	-	-	74.1	94.4	87.0	23.9	64.0	81.3	42.7	11.8
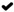	-	-	-	-	74.5	95.1	86.7	24.9	63.8	82.1	48.4	12.8
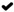	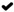	-	-	-	74.8	94.8	87.1	24.3	64.7	81.9	47.4	12.1
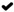	-	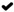	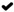	-	74.6	94.7	87.1	24.0	64.4	81.7	112.3	26.0
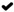	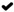	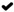	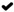	-	75.2	94.9	87.9	24.2	64.5	82.7	111.4	25.3
-	-	-	-	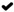	75.3	95.3	87.7	26.1	64.7	82.5	42.7	11.8
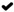	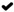	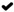	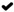	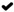	75.9	95.8	89.8	28.0	65.2	83.1	111.4	25.3

**Table 3:** Ablation study on the MarShipInsSeg dataset.

CVTA	DOWconv	SIoU	CBAM	Mixup	AP (%)	AP_50_ (%)	AP_75_ (%)	AP_S_ (%)	AP_M_ (%)	AP_L_ (%)
-	-	-	-	-	46.6	81.0	47.6	27.1	50.4	61.1
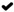	-	-	-	-	47.1	81.5	48.4	27.0	50.3	62.4
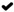	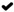	-	-	-	46.9	80.7	48.0	27.4	50.2	61.1
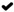	-	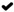	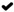	-	47.0	81.7	48.2	27.6	49.9	61.3
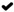	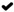	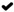	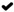	-	47.6	81.7	50.1	27.9	51.0	62.6
				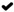	48.8	84.2	50.9	28.1	52.3	64.4
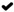	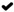	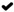	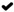	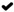	49.3	84.3	51.7	28.6	52.3	65.2

**Table 4:** Effect of MixUp α on VLRSSD performance.

α	AP (%)	AP_50_ (%)	AP_75_ (%)	AP_S_ (%)	AP_M_ (%)	AP_L_ (%)
0.3	74.3	95.1	87.5	18.4	64.0	81.6
8.0	75.8	95.5	89.2	26.5	65.3	83.0
16.0	75.3	95.8	87.9	25.0	65.2	82.4
24.0	75.8	95.8	88.5	23.5	64.9	83.2
32.0	75.9	95.8	89.8	28.0	65.2	83.1
36.0	76.0	95.6	89.3	24.1	65.7	83.1

**Table 5:** Effect of CVTA replacement on VLRSSD.

Replaced Stages	AP (%)	AP_50_ (%)	AP_75_ (%)	AP_S_ (%)	AP_M_ (%)	AP_L_ (%)
1	75.1	95.2	88.6	25.3	63.8	82.3
2	75.3	95.4	88.6	24.7	64.8	82.4
2,3	75.9	95.8	89.8	28.0	65.2	83.1
3,4	75.6	95.6	89.4	23.6	65.4	82.7
1,2,3	75.4	95.5	88.4	26.7	64.7	82.6
1,2,3,4	75.9	95.7	88.9	26.1	65.5	83.1
More	OOM					

**Table 6:** Comparative experiments on the VLRSSD dataset.

Methods	AP (%)	AP_50_ (%)	AP_75_ (%)	AP_S_ (%)	AP_M_ (%)	AP_L_ (%)
Mask R-CNN [16]	72.0	92.5	84.8	23.2	61.2	79.0
YOLACT [26]	60.2	85.2	68.9	17.1	47.6	68.0
HTC [60]	74.8	93.4	86.6	21.3	63.5	82.3
SOLO [24]	27.7	48.5	29.0	0.1	12.9	36.3
SOLOv2 [25]	58.7	84.2	66.1	2.1	43.9	68.8
Cascade Mask R-CNN [18]	73.0	91.3	84.9	18.0	62.3	80.1
Mask2former [61]	72.2	90.1	82.0	12.1	59.0	81.4
Oneformer [62]	73.1	92.3	84.5	18.3	62.2	79.8
Mask DINO [63]	74.5	94.3	86.9	24.2	64.8	81.4
CATNet [19]	71.4	91.1	82.7	22.7	59.8	79.0
YOLOv11 [64]	74.4	94.9	87.2	25.4	63.4	81.9
CDSANet (ours)	75.9	95.8	89.8	28.0	65.2	83.1

**Table 7:** Comparative experiments on the MarishipInSseg dataset.

Methods	AP (%)	AP_50_ (%)	AP_75_ (%)	AP_S_ (%)	AP_M_ (%)	AP_L_ (%)
Mask R-CNN	45.8	79.0	48.4	25.4	47.4	62.5
YOLACT	42.4	77.2	42.0	22.5	42.6	59.2
SOLO	26.0	46.7	26.4	5.5	24.7	47.6
SOLOv2	39.7	70.9	39.2	17.5	40.4	59.5
Cascade Mask R-CNN	46.0	78.6	48.9	25.5	46.4	63.5
HTC	46.6	80.9	48.2	25.8	47.9	63.4
CATNet	46.0	80.5	47.5	26.7	47.8	60.8
YOLOv11	46.9	81.6	48.7	27.4	49.9	62.0
CDSANet (ours)	49.3	84.3	51.7	28.6	52.3	65.2

**Table 8:** Other evaluation metrics for different models.

Methods	AP_T (%)	AP_M (%)	AP_W (%)	AP_S (%)	AP_Md (%)	AP_D (%)	B-AP (%)	B-AP_50_ (%)	F-B (%)
Baseline	71.3	72.0	77.0	78.0	71.2	70.3	67.9	94.0	95.9
HTC	73.8	70.7	78.8	79.5	71.3	70.0	68.2	92.5	94.4
CATNet	70.5	66.9	76.4	77.6	67.1	63.9	65.5	90.2	92.2
YOLOv11	72.1	71.7	77.7	78.2	71.4	71.3	68.5	94.5	96.4
CDSANet (ours)	73.0	73.7	79.1	79.4	72.7	74.8	69.9	95.7	97.3

**Table 9:** Comparison of model parameters.

Methods	Param (M)	FLOPs (G)	FPS	Latency (ms)
Mask R-CNN [16]	63.0	149.0	68.9	14.5
YOLACT [26]	53.7	112.0	93.4	10.7
SOLO [24]	55.1	148.0	54.4	18.4
SOLOv2 [25]	65.2	146.0	63.1	15.8
Cascade Mask R-CNN [18]	96.0	1667.0	47.7	21.0
HTC [18]	96.1	1589.0	49.6	20.2
CATNet [19]	73.6	127.0	30.4	32.9
Baseline	11.8	42.7	197.6	5.1
CDSANet (RTX4090)	25.3	111.4	130.3	7.7
CDSANet (RTX4070)	-	-	38.5	26.0

**Table 10:** Error analysis results on VLRSSD datasets.

Method	C_50_ (%)	C_75_ (%)	Loc (%)	Sim (%)	Other (%)	BG (%)	FN (%)
Baseline [11]	94.4	87.0	1.4	0.0	0.0	3.2	1.0
CATNet [19]	91.1	82.7	2.1	0.0	0.0	1.8	5.0
HTC [18]	93.4	86.6	1.6	0.0	0.0	2.0	3.0
CDSANet	95.8	89.8	1.0	0.0	0.0	2.2	1.0

## Data Availability

Data are publicly available at https://github.com/RainbowSugar1/Datasets (accessed on 26 October 2025).
